# Hydroxyproline in Urine Microvesicles as a Biomarker of Fibrosis in the Renal Transplant Patient

**DOI:** 10.3390/biomedicines12122836

**Published:** 2024-12-13

**Authors:** María José Torres Sánchez, María Carmen Ruiz Fuentes, Elena Clavero García, Noelia Rísquez Chica, Karla Espinoza Muñoz, María José Espigares Huete, Mercedes Caba Molina, Antonio Osuna, Rosemary Wangensteen

**Affiliations:** 1 Nephrology Department, Hospital Universitario Virgen de las Nieves, Instituto de Investigación Biosanitaria, Ibs Granada, University of Granada, 18071 Granada, Spain; m.torres.sanchez.sspa@juntadeandalucia.es (M.J.T.S.); elenaclaverogarcia@gmail.com (E.C.G.); noelia.rc.9295@gmail.com (N.R.C.); karlae_21@hotmail.com (K.E.M.); mjose.espigares.sspa@juntadeandalucia.es (M.J.E.H.); 2 Department of Medicine, University of Granada, 18071 Granada, Spain; aosunaortega@ugr.es; 3 Department of Pathological Anatomy, Provincial Unit of Pathological Anatomy of Granada (UPIGAP), Instituto de Investigación Biosanitaria, Ibs Granada, University of Granada, 18071 Granada, Spain; mercedes.caba.sspa@juntadeandalucia.es; 4 Area of Physiology, Department of Health Sciencies, University of Jaen, 23071 Jaen, Spain; rwangens@ujaen.es

**Keywords:** biomarker, hydroxyproline, kidney, microvesicles, transplantation

## Abstract

**Background/Objectives**: Interstitial fibrosis/tubular atrophy in kidney transplantation is an unspecific lesion induced by immune and non-immune factors, which determines the progression of chronic kidney disease. Hydroxyproline is an imino acid that is part of the molecule of collagen. The aim of this study was to assess hydroxyproline in urine microvesicles as a marker of fibrosis in the renal transplant patient. **Patients and Methods**: An observational cross-sectional study was conducted on 46 renal transplant patients who had undergone renal biopsy with diagnostic intention, as well as 19 healthy controls. Clinical, histological, and laboratory variables were collected at the time of marker determination and renal function was analyzed 2 years later. Hydroxyproline was measured in urine microvesicles. **Results**: Renal transplant patients showed a higher microvesicular concentration of hydroxyproline compared to the control group, with the following medians (interquartile range (IQR)): 28.024 (5.53) ng/mL vs. 2.51 (1.16) ng/mL, *p* < 0.001. In the transplanted patients, patients in whom biopsy showed some score of total cortical parenchymal inflammation (ti) displayed a significantly higher concentration of hydroxyproline in urine microvesicles than those patients who did not score for cortical parenchymal inflammation (29.91 ± 2.797 ng/mL vs. 22.72 ± 8.697 ng/mL, *p* = 0.034). No significant correlation was observed between urinary markers and serum creatinine, calcium, and parathyroid hormone (PTH). **Conclusions**: The concentration of hydroxyproline in urinary microvesicles increased in renal transplant patients relative to healthy controls. Hydroxyproline in urinary microvesicles is a marker of chronic renal inflammation in transplanted patients, and further studies are required to confirm this finding in other pathologies, as well as the association with fibrosis and the evolution of renal function.

## 1. Introduction

Although post-renal transplant results have improved in recent decades, renal graft loss remains a major clinical problem. The decrease in the rate of short-term graft loss, due to improved immunosuppression, as well as the prevention of acute rejection, does not translate into better long-term renal graft survival [1].

Interstitial fibrosis together with tubular atrophy (IFTA) are the responses to virtually any sustained renal damage and inversely correlate with renal function and graft survival [2].

Renal fibrosis can affect all renal compartments, glomerular (glomerulosclerosis), tubulointerstitial and vascular (arterial and arteriolosclerosis). Tubulointerstitial fibrosis is characterized by an excessive accumulation of extracellular matrix proteins. Known factors that cause interstitial fibrosis (IF) in kidney transplantation are mediated by the immune system or non-immune system, including acute and chronic rejection, high blood pressure, dyslipidemia, diabetes mellitus, infections, ischemia, and immunosuppressive agents, particularly calcineurin inhibitors [3].

Tubulointerstitial fibrosis is the common end result of most progressive kidney diseases and the most powerful predictor of renal survival [2,3,4,5]. In renal allografts, it is evaluated together with tubular atrophy and is referred to as interstitial fibrosis/tubular atrophy (IFTA) [6]. IFTA is detectable in approximately 40% of renal allografts at 3–6 months [7,8] and increases to approximately 65% at 2 years [8]. IFTA is a non-specific lesion and an independent risk factor for delayed graft loss, particularly in expanded criteria donor grafts, regardless of whether or not an allograft-specific disease was diagnosed [9,10].

IFTA, especially when accompanied by inflammation, correlates with lower graft survival [11,12]. Graft survival at 10 years has been reported to be 95% in patients without IFTA, but only 41% in patients with IFTA and transplant vasculopathy [13].

Renal biopsy remains the gold standard for essential diagnostic and prognostic information after renal transplantation [14]. In addition to the indication biopsies, some groups also perform protocol biopsies as part of their follow-up program. Protocol biopsies are useful for detecting chronic subclinical diseases and for tracking the progression of renal fibrosis [15,16].

Banff’s classification is the standard in the evaluation of renal allograft biopsies and allows for a good prediction of graft survival. Renal biopsy is an invasive method that is not free of complications, the most common being bleeding, so it is desirable to have noninvasive biomarkers for diagnosis. Biomarkers can help predict the long-term outcome of the graft or differentiate between chronic rejection and IF/TA of another origin.

Collagen is a major component of animal tissues. Hydroxyproline is an imino acid that is traditionally used as a collagen deposition index [17]. Its presence has been observed in urinary microvesicles from the kidney tubular epithelium.

The main objective of this study was to assess hydroxyproline in urine microvesicles as a marker of fibrosis in the renal transplant patient. To do this, we analyzed the relationship between hydroxyproline concentration in urine microvesicles with the presence of IFTA in renal transplantation, the presence of inflammatory findings on renal biopsy, and known clinical factors in the development of fibrosis in the renal transplant patient.

## 2. Patients and Methods

### 2.1. Study Design

We conducted a cross-sectional observational study of 46 renal transplant patients at Virgen de las Nieves University Hospital between 2013 and 2022 who had undergone renal graft biopsy with diagnostic intent, as well as 19 healthy controls.

In transplanted patients, age, sex, type of donation, cold ischemia time, anticalcineurinic treatment, treatment with renin–angiotensin–aldosterone-blocking agents, recipient cardiovascular diseases such as hypertension, post-transplantation diabetes mellitus, cytomegalovirus (CMV) infection, and polyomavirus bk were collected. Graft loss and patient exitus were recorded. Histological data were collected as classified by Banff: presence of glomerulitis (g), tubulitis (t), interstitial inflammation (i), arteritis (v), peritubular capillaritis (ptc), extent of total cortical inflammation (ti), presence of plasma cells (10%), interstitial fibrosis and tubular atrophy (IFTA), C4d staining, transplant glomerulopathy (cg), and histological diagnosis. The following analytical parameters were recorded: baseline serum creatinine at biopsy, creatinine per year and at 2 years of diagnostic test, calcium, phosphorus, and PTH in blood. In both transplant patients and the healthy controls, urine creatinine, protein, and hydroxyproline in urinary microvesicles were determined.

An estimated glomerular filtration rate (eGFR) drop using CKD-EPI, greater than 5 mL/min/m^2^ per year (as defined by the KDIGO guidelines), was considered to be impaired in renal function.

### 2.2. Processing of Urine Samples

Urine samples were centrifugated just after collection at 1000× *g*, 15 min, 4 °C in order to separate whole cells, bacteria, cellular debris, and other substances present in the urine. Precipitates were discarded. Some aliquots of supernatants were frozen at −80 °C. An amount of 10 mL of each supernatant was distributed in five Eppendorf tubes and subjected to a second centrifugation step at 17,000× *g*, 20 min, 4 °C. Precipitates including microsomal fraction (microvesicles, ectosomes, and large membrane fragments) were frozen at −80 °C until analysis. On the day of analysis, 40 µL of saline serum was added to each sample, shaking for 2 min to obtain a total of 200 µL of resuspended sample that contained 50-fold concentrated microsomal fraction in relation to the original urine sample.

### 2.3. Analytical Procedures

Creatinine was measured in serum and urine samples using an autoanalyzer Spin120. The reagent for the creatinine Jaffé method (ref. MI1001111) was provided by Spinreact (Barcelona, Spain).

In microsomal fractions, hydroxyproline was quantified using an ELISA kit purchased from Sunlong Biotech (Hangzhou, China), and total protein content was determined in a 96-well microplate using a QuantiPro BCA assay kit from Sigma-Aldrich (St. Louis, MO, USA). The content of hydroxyproline in a microsomal fraction was standardized by concentration in urine, urinary creatinine concentration, and total protein content of the microsomal fraction.

Descriptive statistical analysis was performed where quantitative variables were expressed as mean ± standard deviation (sd) or median (interquartile range (IQR)) according to their distribution and categorical variables using frequency table and percentages. The normality of the variables was checked with the Kolmogorov–Smirnov test in order to determine whether parametric or non-parametric tests would be used later.

In addition, in order to compare the results between patient groups against quantitative variables, Student’s *t* test was used for independent samples, or Welch’s for analysis of variance if distributed according to normal distribution; otherwise, the Mann–Whitney U-test or Kruskall–Wallis test was used. In addition, Spearman’s correlation was used to determine the relationship between quantitative variables.

Subsequently, a linear mixed effects model was carried out to study the evolution of creatinine over two years; the fixed effects component was initially timed and then adjusted for urinary hydroxyproline; comparisons of two-to-two of the estimated marginal averages for the time variable with Bonferroni correction were made. A repeat measurement study using parametric methods (ANOVA) and non-parametric methods (Friedman) was carried out according to the distribution of the variable in each case.

The level of significance considered for all contrasts was 0.05. The software used for statistical analysis was STATA version 16 and SPSS Statistics 20.

## 3. Results

### 3.1. Demographic Study

In our cohort, 71.74% (n = 33) of renal transplant recipients were male and 28.26% (n = 13) were women, with a mean age of 45.61 ± 14.88 years. The most common CKD etiology was glomerular at 30.43% (n = 14), followed by unknown cause at 19.57% (n = 9), and interstitial at 17.39% (n = 8) as the first three causes. The characteristics of the donor and donation process are as follows: donor age 47.65 ± 15.12 years, cold ischemia time 13 ± 5.59 h, men 63.04%, live donor 6.52%, brain death 56.52%, uncontrolled donation after cardiac death 10.87%, and controlled donation after cardiac death 23.91%. Immunosuppressive induction treatment was used in 75.5% of cases (basiliximab 32.35%, thymoglobulin 67.65%). Patients had hypertension and pretransplant diabetes mellitus in 86.96% and 8.7% of cases, respectively. Post-transplant diabetes mellitus developed in 13.04% of patients. A total of 91.3% of patients on anticalcineurinic therapy were treated with tacrolimus, and 8.7% were treated with ciclosporin; 97.83% were taking mycophenolate, 13.04% mTOR inhibitor, and 100% prednisone. Immunosuppressive therapy was altered in 21.74% of patients at some time during the period of transplant. Treatment with a renin–angiotensin system blockade was prescribed in 34.78% of patients, while 10.87% were on antialdosterone therapy. A total of 6.52% had a histologic diagnosis of cell active rejection, and 19.57% had antibody-mediated rejection (acute or chronic). Regarding infections, 39.13% had an episode of urinary tract infection, CMV 45.65%, and BK infection 15.22%. Table 1 shows these data.

The histological characteristics are shown in Table 2.

### 3.2. Comparison of Transplant Patients with Control Group

The comparative analysis of urinary markers was initially conducted between case groups (renal transplantation) and control groups (Table 3). There were significant differences in all urinary markers, with higher levels of microvesicle hydroxyproline concentration in renal transplant patients compared to the control group: 28.024 (5.53) ng/mL vs. 2.51 (1.16) ng/mL, *p* < 0.001.

### 3.3. Transplanted Patients Study

Bivariate analyses were performed in the group of renal transplant patients (cases) to study the presence of differences in urinary markers with respect to clinical and histological variables collected in this study. Furthermore, correlation tests and evolution studies in renal function were carried out.

#### 3.3.1. Comparison of Markers in Relation to Clinical Characteristics

Regarding the cases and clinical variables, such as recipient sex, HTA and DM pre-transplant, post-transplant diabetes, induction treatment, CMV infection, BK infection, renin blockade, and angiotensin therapy, there were no significant differences in the concentration of urinary markers according to the presence or absence of the characteristic studied.

#### 3.3.2. Comparison of Urinary Markers Relative to Histological Characteristics

In terms of the presence or absence of any lesion score according to the pathological characteristics of the Banff classification, patients who had some degree of inflammation throughout the cortical parenchyma (extent of total cortical inflammation “ti”) on biopsy had significantly higher levels of hydroxyproline in total urine microvesicles than those who did not (29.91 ± 2.797 ng/mL vs. 22.72 ± 8.697 ng/mL, *p* = 0.034) (Table 4). There were no significant differences in hydroxyproline concentration in relation to microvesicular protein concentration and hydroxyproline relative to urinary creatinine.

We did not find significant differences in the rest of the Banff parameters.

**Figure 1:**
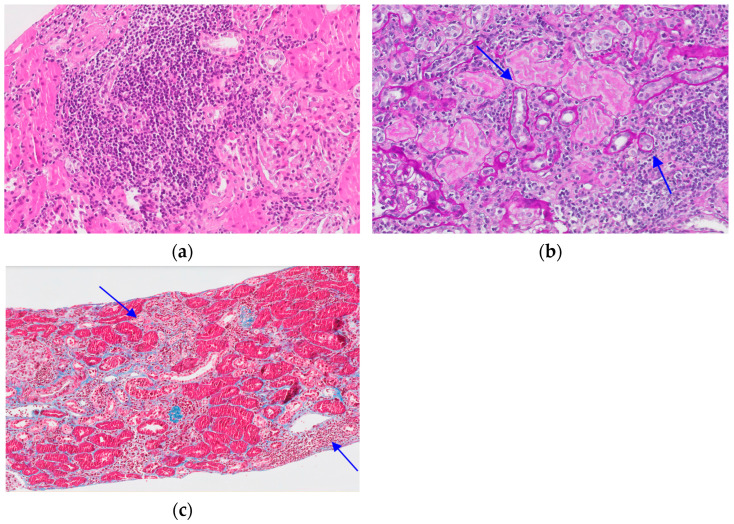
Images of inflammatory infiltrate “ti” of one biopsy sample. (**a**) Interstitial inflammatory infiltrate. H-E × 20. (**b**) Areas of tubular atrophy associated with inflammatory infiltrate. PAS × 10. (**c**) Areas of interstitial fibrosis associated with inflammatory infiltrate. Masson’s trichrome × 10. The arrows refer to the explanation of each image.

Images of the presence of “ti” in a study patient are shown in Figure 1.

Studying the differences in the presence of “ti” in the fourth quartile of hydroxyproline concentration in our sample of patients, we found significant differences, with a higher concentration of hydroxyproline in patients with “ti” compared to those without this characteristic. The study of positive and negative predictive values, as well as the sensitivity and specificity of this urinary marker in relation to lesion presentation, is reflected in Table 5.

#### 3.3.3. Correlation Study of Urinary Markers, Serum Creatinine, and Parameters of Bone Mineral Metabolism

No significant correlation was observed between urinary markers and serum creatinine (r(Spearman’s rho) = 0.032, *p* = 0.98), phosphorus (r = 0.19, *p* = 0.22), calcium (r = −0.18, *p* = 0.23), and PTH (r = 0.096, *p* = 0.53).

#### 3.3.4. Renal Function Evolution Study Following Urinary Marker Determination

Serum creatinine increased significantly in all patients within two years of the determination of hydroxyproline in urine microvesicles. The baseline creatinine was (SCr_0) 2.05 mg/dL ± 0.86 mg/dL, creatinine per year was (SCr_1) 2.01 ± 0.68 mg/dL, and creatinine at two years was (SCr_2) 2.31 ± 1.17 mg/dL, *p* = 0.026 (SCr_0 vs. SCr_2, *p* = 0.05; SCr_1 vs. SCr_2, *p* = 0.008).

**Figure 2:**
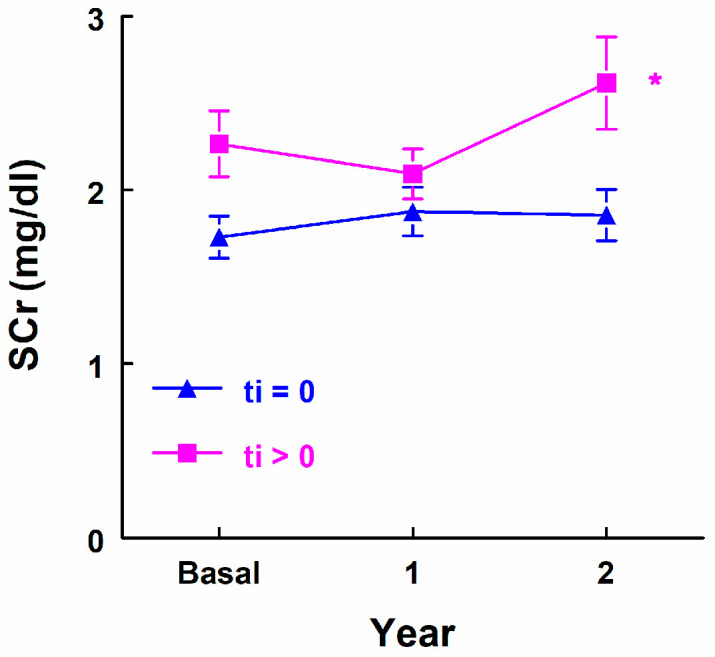
Serum creatinine levels in all patients, in relation to ti = 0 vs. ti > 0. * Statistical significance intragroup.

In relation to whether there was a presence of “ti”, there were no significant differences in the evolution of serum creatinine in the two subsequent years (*p* = 0.084). In the ti = 0 group, there was no significant difference; however, a significant difference was observed in the group with ti > 0 (*p* = 0.042) with differences between SCr_0 and SCr_2 (*p* = 0.043), and between SCr_1 and SCr_2 (*p* = 0.005) after the determination of hydroxyproline (Figure 2).

**Figure 3:**
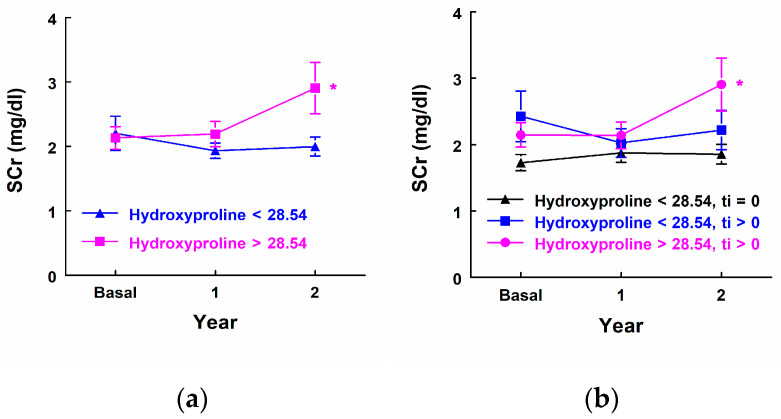
Comparison of serum creatinine levels between the highest hydroxyproline quartile group and the lower quartiles. (**a**) Serum creatinine levels in patients within the highest quartile of hydroxyproline concentration (ng/mL) compared to those in the lower quartiles. (**b**) Serum creatinine levels in patients within the highest quartile of hydroxyproline concentration (ng/mL) compared to the remaining quartiles, stratified by ti = 0 or ti > 0. * Indicates statistically significant intragroup differences.

Comparing the highest hydroxyproline quartile (>28.54 ng/mL) relative to the rest of the lower quartiles, there were no significant differences in the evolution of serum creatinine in the two subsequent years, but it was close to significant (*p* = 0.059) (Figure 3a). In the group of patients with hydroxyproline concentration in the highest quartile (all with ti > 0), a significant increase in serum creatinine was observed within two years (*p* = 0.013) (SCr_0 vs. SCr_2, *p* = 0.021; SCr_1 vs. SCr_2, *p* = 0.012) (Figure 3b). In the remaining quartiles with lower hydroxyproline concentration, no significant difference in serum creatinine was observed over time in the group with ti = 0, and neither in the group with ti > 0. (Figure 3b).

A logistic regression model could not be performed due to the limitation of sample size.

## 4. Discussion

The main finding of this study is the detection of increased levels of hydroxyproline in urinary microvesicles in renal transplant patients compared to healthy controls. In addition, in transplanted patients with inflammation throughout the cortical parenchyma on the renal graft biopsy, a significantly higher concentration of hydroxyproline in urinary microvesicles was found than in patients without this finding.

Tubulointerstitial fibrosis in the kidney is the ultimate consequence of an active and chronic inflammatory stimulus of any etiology on that organ. In the case of renal transplantation, factors related to the development of interstitial fibrosis may be immune-mediated or non-immune-mediated [18]. The relationship with the immune and antibody-mediated responses has been described thanks to the transcriptomics carried out in renal transplant biopsies—studies suggesting that the deterioration of renal function is more related to continued injury to the nephron than to fibrogenesis [19]. Transplant patients in whom immune tolerance is achieved do not show inflammation, tubulointerstitial fibrosis, or arteriolar hyalinosis in renal biopsy [20].

Other influencing factors of epithelial–mesenchymal transition include sustained immunosuppression (mainly calcineurin inhibitors from the first year post-transplantation), arterial hypertension, dyslipidemia, diabetes mellitus, infections, and preimplantation inflammation in the donated organ, secondary to hypoperfusion or ischemia–reperfusion damage [18].

The degree of interstitial fibrosis correlates with renal function and tubular atrophy and is considered a single endpoint—interstitial fibrosis and tubular atrophy (FIAT).

Renal biopsy is the “gold-standard” for the definitive diagnosis of the presence of tubulointerstitial fibrosis and its precursor inflammatory lesions, which are systematized in the Banff classification [21]. Other earlier urinary or systemic markers than routine clinical markers (estimated glomerular filtration rate, proteinuria, or hematuria) that are more accurate than biopsy are continuously researched. These biomarkers would allow for anticipation in the diagnosis and in diagnosis by biopsy, which, although probably being able to offer a definitive diagnosis, is an invasive method, as well as late [18]. There are even ultrasound or magnetic nuclear resonance studies, which attempt to link radiological findings to the extent of fibrosis in the renal graft [22].

The study of biomarkers of kidney damage has grown significantly thanks to omics, microRNA, DNA-free, etc. But most of them are not organ-specific.

Hydroxyproline is an imino acid derived from proline residues, which account for 20% of all collagen amino acids, and half of which are hydroxylated. Collagen is one of the main components of the increased extracellular matrix leading to fibrosis [23].

The determination of urinary and renal hydroxyproline has been performed in several experimental studies, looking at the relationship of its presence in urine to the development of renal injury and fibrosis, with various results. Thus, in a murine nephrotoxicity study produced by melanin and cyanuric acid [24], a relationship between high urinary hydroxyproline levels and renal fibrosis was observed in 344 rats. In another subsequent study [25] in obese Zucker rats, urinary hydroxyproline and renal tissue were determined, together with amino peptidases and Klotho. In rats aged 2 months, urinary hydroxyproline was increased, and at 8 months, it was related to the presence of glomerular sclerosis, glomerular cysts, and inflammation, but not to fibrosis or renal hydroxyproline content.

In clinical studies, the determination of urinary hydroxyproline was found mainly in relation to bone pathology, e.g., osteoporosis in Parkinson’s disease [26], in which urine hydroxyproline values are increased in relation to controls without osteoporosis, or in tumors of dental origin [27]. There are few studies relating the presence of collagen derivatives in urine in large patient series and their correlation with renal function [28], as well as bone metabolism [29].

In our study, the aim was to study the relationship of hydroxyproline in urinary microvesicles, as a marker derived from collagen and fibrosis to anatomopathological findings, in biopsies in renal transplant patients with diagnostic intention. The increase in microvesicular hydroxyproline in the cases related to the controls could initially suggest, as published, an increase in inflammation or fibrosis in such patients.

In transplant patients, significant differences were found in the determination of microvesicular hydroxyproline in urine, with greater concentration in those patients with inflammation throughout the cortical parenchyma (ti) versus those who did not have it. The “ti” lesion (total inflammation according to Banff’s classification [21]) refers to inflammation throughout the cortical parenchyma, including areas of interstitial fibrosis and tubular atrophy, the subcapsular cortex, and the perivascular cortex. However, when we looked at the correlation of microvesicular hydroxyproline in urine with the histologic presence of interstitial fibrosis and tubular atrophy, we found no significant difference. There are studies describing, with the exception of antibody-mediated rejection, that all Banff lesion categories show lower or equal sensitivity and specificity (including FIAT) to predict graft survival compared to "ti” [30]. These findings are consistent with the Montoro-Molina rat experimental study [25], in which Klotho and aminopeptidases were related to scarring over time, hydroxyproline with inflammation and sclerosis, and, later, fibrosis. The relationship described in our current study may reflect that microvesicular hydroxyproline may be a marker of chronic active inflammation prior to the development of fibrosis and prognosis of renal graft.

In the literature, the determination of urinary hydroxyproline in renal transplantation is limited to its potentially significant relationship with bone metabolism and, in particular, bone resorption [31]. In this current study, there was no correlation between microvesicular hydroxyproline and markers of bone metabolism determined in normal clinical practice, which could be explained by the fact that microvesicles present in the urine have an exclusively renal origin and need not be related to bone metabolism variables.

Regarding renal function, in our study, creatinine increased in patients with higher levels of hydroxyproline in urine microvesicles, and all of them with a ti > 0. However, in patients with lower hydroxyproline levels and ti, such a difference was not found; this could be because in patients with lower concentrations with ti, there may be inflammation but not enough inflammatory damage vs. probable fibrosis to trigger increased creatinine, as would be the case in patients with higher hydroxyproline levels.

Extracellular vesicles (EVs), including microvesicles and exosomes, have been demonstrated to be present in urine [32,33] and constitute a promising source of biomarkers for the diagnosis and therapy of renal diseases [34]. The quantitation of microvesicles in urine is not fully standardized. In a previous study of our group using cisplatin-treated rats, we showed that the content of glutamyl aminopeptidase (GluAp), an enzyme derived from tubular epithelium, was increased in microvesicular and exosomal fractions of urine and could serve as an early biomarker of acute kidney injury [35]. In this previous work, microvesicular and exosomal content was expressed by diuresis, creatinine concentration, and total protein content in EVs, showing that GluAp was increased independently of the method of standardization. In this present work, we show that the hydroxyproline content of microvesicles, an index of the content of collagen, is higher in transplanted patients when it is quantified by concentration in urine, creatinine concentration, or microvesicular protein content. Furthermore, we demonstrate that the content of microvesicular hydroxyproline, when it is standardized by concentration in urine, is higher in transplanted patients with total inflammation when compared to patients with no inflammation. Nevertheless, we did not find any significant differences in these patients when hydroxyproline content was expressed as creatinine concentration or microvesicular protein. This can be due to several factors. First, creatinine excretion is an index of glomerular filtration but microvesicles are generated from renal epithelial cells. Therefore, their presence in urine is not necessarily related to creatinine excretion. Further, the inflammation process might affect the amount of microvesicles derived from epithelial cells, altering the total protein content of microvesicular fraction in these patients. Altogether, these results indicate that total urinary concentration is the best method to quantify microvesicular hydroxyproline content when compared with creatinine concentration or microvesicular protein.

The limitations of this study can be summarized in the following points: the hydroxyproline study is limited to cross-sectional cut-off in a limited sample size and with a large margin of time since renal transplantation. The prospective renal function study was for two years, with little time to progress for a slow-moving assessment of fibrosis. More specific bone metabolism studies would be needed to definitively rule out the effect of bone resorption on the elimination of hydroxyproline in urinary microvesicles.

## 5. Conclusions

In conclusion, the determination of hydroxyproline in urinary microvesicles is significantly elevated in transplanted patients relative to the healthy controls, which may indicate an increase in inflammation, and its effect on the evolution of renal fibrosis cannot be ruled out. In our cohort of renal transplant recipients, urine microvesicular hydroxyproline was associated with renal cortical inflammation displaying a “chronicity” profile but not with established FIAT, and the elevation of hydroxyproline in urine microvesicles is a potential marker of renal function deterioration. Therefore, studies with larger sample sizes and prospective follow-ups are needed to determine whether this marker can predict future renal fibrosis or its correlation with renal graft prognosis.

## Figures and Tables

**Table 1:** Demographic study.

Qualitative Demographic Data				
Receptor Sex	Male		Female	
	71.74% (33) *		28.26% (13)	
Etiology	Glomerular	Unknown		Interstitial
	30.43% (14)	19.57% (9)		17.39% (8)
Donation process	Live donor	Brain death		Cardiac death
	6.52% (3)	56.52% (26)		34.78% (16)
Induction treatment	No	Basiliximab		Thymoglobulin
	23.91% (11)	23.91% (11)		50% (23)
	Yes		No	
Diabetes mellitus preTx	8.7% (4)		91.3% (42)	
Hypertension preTx	86.96% (40)		13.04% (6)	
IS change	21.74% (10)		78.26% (36)	
Diabetes mellitus post-Tx	13.04% (6)		86.96% (40)	
ACE inhibitors/ARA II	34.78% (16)		65.22% (30)	
Antialdosteronic therapy	10.87% (5)		89.13% (41)	
Cell active rejection	6.52% (3)		93.48% (43)	
AMR	19.57% (9)		80.43% (37)	
CMV infection	45.65% (21)		54.35% (25)	
BK infection	15.22% (39)		84.78% (39)	
Urinary infections	39.13% (18)		60.87% (28)	
Graft failure	4.35% (2)		95.65% (44)	
**Quantitative demographic data**				
Receptor age	45.61 ± 14.88 **			
Donor age	47.65 ± 15.12			
Cold ischemia time	13 ± 5.59			

* % (n). PreTx: pretransplantation. IS: immunosuppression. Post-Tx: postransplantation. ACE: angiotensin convertasa enzyme. ARA II: angiotensin II receptor antagonists. AMR: antibody-mediated rejection. ** Mean ± standard deviation.

**Table 2:** Histological scores in kidney transplantation group (Banff).

Histological Feature	Score *			
Interstitial Inflammation (i)	i0	i1	i2	i3
	63.04% (29)	19.57% (9)	0%	2.17% (1)
Tubulitis (t)	t0	t1	t2	t3
	39.13% (18)	23.91% (11)	8.7% (4)	0%
Arteritis (v)	v0	v1	v2	v3
	54.35% (1)	2.17% (1)	2.17% (1)	0%
Glomerulitis (g)	g0	g1	g2	g3
	43.48% (20)	17.39% (8)	10.87% (5)	13.04% (6)
Peritubular Capillaritis (ptc)	ptc0	ptc1	ptc2	ptc3
	58.70% (27)	8.7% (4)	13.04% (6)	2.17% (1)
Total Inflammation (ti)	ti0	ti1	ti2	ti3
	39.13% (18)	30.43% (14)	2.17%(1)	0%
Interstitial fibrosis and tubular atrophy (IFTA)	IFTA 0	IFTA 1	IFTA 2	IFTA 3
	30.43% (14)	26.09% (12)	36.96% (17)	2.17% (1)
C4d	C4d0	C4d1	C4d2	C4d3
	76.09% (35)	13.04% (6)	6.52% (3)	0%
Glomerular Basement Membrane Double Contours (cg)	No (cg0)		Yes (cg > 0)	
	89.13% (41)		10.87% (5)	

* Score. i0: 10%; i1: 10–25%; i2: 26–50%; i3: >50%. t0: without inflammatory cells; t1: one to four cells/tubular section; t2: five to ten cells/section; t3: >10 cells/section. v0: no arteritis; v1: intimal arteritis in 25% of lumen; v2: intimal arteritis ≥ 25% de la luz in ≥one artery; v3: transmural arteritis with/without fibrinoid necrosis. g0: Non-glomerulitis; g1: 25%; g2: 25–75%; g3: >75%. ptc0: 10% > two cells/PTC; ptc1: >10% three to four cells/PTC; ptc2: >10% five to ten cells/PTC; ptc3: >10% >10 cells/PTC. ti0: 10%; ti1: 10–25%; ti2: 26–50%; ti3: >50%. IFTA 0: <10%; 1: 10–25%; 2: 26–50%; 3: >50%. C4d: staining for C4d on endothelial cells of PTCs and medullary vasa recta; C4d0: 0%; C4d1: 1–9%; C4d2: 10–50%; C4d3: >50%.

**Table 3:** Comparison of urine microvesicular hydroxyproline concentration between case and control groups.

Marker	Case (n = 45)	Control (n = 18)	*p*-Value
Microvesicular hydroxyproline in urine (ng/mL)	28.024 (5.53) *	2.51 (1.16)	*p* < 0.001
	25.200 ± 0.99 **	2.61 ± 0.897	
Hydroxyproline/microvesicular protein (ng/mg)	1136.818 (1136.82) *	84.595 (104.14)	*p* < 0.001
	1845.031 ± 2052 **	118.83 ± 150.85	
Hydroxyproline/Creatinine in urine (ng/mg)	34.72 (36.54) *	3.19 (3.69)	*p* < 0.001
	45.38 ± 30.54 **	4.25 ± 3.09	

* Median (interquartile range (IQR)); ** Mean ± standard deviation (SD).

**Table 4:** Comparison of urine microvesicular hydroxyproline concentration in cases with or without inflammation of the total parenchyma (ti).

Marker	ti	Mean ± SD *	Median (IQR) **	*p*-Value
Microvesicular hydroxyproline in urine (ng/mL)	No (18)	22.72 ± 8.697	27.12 (7.04)	*p* = 0.034
	Yes (13)	29.91 ± 2.797	31.298 (3.84)	
Hydroxyproline/microvesicular protein (ng/mg)	No (18)	2301.29 ± 4243.14	883.64 (986.13)	*p* = 0.522
	Yes (13)	1725.06 ± 1657.08	1642.18 (1922.24)	
Hydroxyproline/Creatinine in urine (ng/mg)	No (18)	37.71 ± 22.49	30.03 (36.03)	*p* = 0.275
	Yes (13)	46.47 ± 21.03	45.83 (37.02)	

* Mean ± standard deviation (SD); ** Median (interquartile range (IQR)).

**Table 5:** Characteristics of hydroxyproline in urine microvesicles test in relation to the presence of “ti” in graft biopsy.

Marker	COV	PPV (%)	NPV (%)	Sens (%)	Spec (%)
Hydroxyproline (ng/mL)	29.83	100%	72.5%	60.71%	100%

Cut-off value (COV), predictive positive value (PPV, %), predictive negative value (PNV, %), sensitivity (Sens, %), specificity (Spec, %).

## Data Availability

The data presented in this study will be available upon request to the corresponding author.

## References

[1] Lamb K.E., Lodhi S., Meier-Kriesche H.-U. (2011). Long-term renal allograft survival in the United States: A critical reappraisal. Am. J. Transplant..

[2] Cieślik A., Burban A., Gniewkiewicz M., Gozdowska J., Dęborska-Materkowska D., Perkowska-Ptasinska A., Kosieradzki M., Durlik M. (2023). The Importance of 1-Year Protocol Biopsy in the Long-Term Prognosis of Kidney Transplants—5-Years Follow-Up. Transplant. Proc..

[3] Miura Y., Satoh S., Saito M., Numakura K., Inoue T., Obara T., Tsuruta H., Narita S., Horikawa Y., Tsuchiya N. (2011). Factors increasing quantitative interstitial fibrosis from 0 hr to 1 year in living kidney transplant patients receiving tacrolimus. Transplantation.

[4] Berchtold L., Ponte B., Moll S., Hadaya K., Seyde O., Bachtler M., Vallée J.-P., Martin P.-Y., Pasch A., de Seigneux S. (2016). Phosphocalcic Markers and Calcification Propensity for Assessment of Interstitial Fibrosis and Vascular Lesions in Kidney Allograft Recipients. PLoS ONE.

[5] Bohle A., Mackensen-Haen S., Gise H.V. (1987). Significance of tubulointerstitial changes in the renal cortex for the excretory function and concentration ability of the kidney: A morphometric contribution. Am. J. Nephrol..

[6] Roufosse C., Simmonds N., Groningen M.C.-V., Haas M., Henriksen K.J., Horsfield C., Loupy A., Mengel M., Perkowska-Ptasińska A., Rabant M. (2018). A 2018 Reference Guide to the Banff Classification of Renal Allograft Pathology. Transplantation.

[7] Heilman R.L., Smith M.L., Kurian S.M., Huskey J., Batra R.K., Chakkera H.A., Katariya N.N., Khamash H., Moss A., Salomon D.R. (2015). Transplanting Kidneys from Deceased Donors with Severe Acute Kidney Injury. Am. J. Transplant..

[8] Loupy A., Vernerey D., Tinel C., Aubert O., Duong van Huyen J.P., Rabant M., Verine J., Nochy D., Empana J.P., Martinez F. (2015). Subclinical Rejection Phenotypes at 1 Year Post-Transplant and Outcome of Kidney Allo-grafts. J. Am. Soc. Nephrol..

[9] Nankivell B.J., Borrows R.J., Fung C.L.-S., O’Connell P.J., Allen R.D., Chapman J.R. (2003). The natural history of chronic allograft nephropathy. N. Engl. J. Med..

[10] Naesens M., Kuypers D.R., De Vusser K., Evenepoel P., Claes K., Bammens B., Meijers B., Sprangers B., Pirenne J., Monbaliu D. (2014). The histology of kidney transplant failure: A long-term follow-up study. Transplantation.

[11] Mannon R.B., Matas A.J., Grande J., LeDuc R., Connett J., Kasiske B., Cecka J.M., Gaston R.S., Cosio F., Gourishankar S. (2010). Inflammation in areas of tubular atrophy in kidney allograft biopsies: A potent predictor of allograft failure. Am. J. Transplant..

[12] Park W.D., Griffin M.D., Cornell L.D., Cosio F.G., Stegall M.D. (2010). Fibrosis with inflammation at one year predicts transplant functional decline. J. Am. Soc. Nephrol..

[13] Serón D., Moreso F., Ramón J.M., Hueso M., Condom E., Fulladosa X., Bover J., Gil-Vernet S., Castelao A.M., Alsina J. (2000). Protocol renal allograft biopsies and the design of clinical trials aimed to prevent or treat chronic allograft nephropathy1. Transplantation.

[14] Colvin R.B., Chang A. (2011). Diagnostic Pathology: Kidney Diseases.

[15] Mehta R., Sood P., Hariharan S. (2016). Subclinical Rejection in Renal Transplantation: Reappraised. Transplantation.

[16] Huang Y., Farkash E. (2016). Protocol Biopsies: Utility and Limitations. Adv. Chronic Kidney Dis..

[17] Hofman K., Hall B., Cleaver H., Marshall S. (2011). High-throughput quantification of hydroxyproline for determination of collagen. Anal. Biochem..

[18] Saritas T., Kramann R. (2021). Kidney Allograft Fibrosis: Diagnostic and Therapeutic Strategies. Transplantation.

[19] Halloran P.F., Famulski K.S., Reeve J. (2016). Molecular assessment of disease states in kidney transplant biopsy samples. Nat. Rev. Nephrol..

[20] Schwarz C., Lawitschka A., Böhmig G.A., Dauber E.M., Greinix H., Kozakowski N., Mühlbacher F., Berlakovich G.A., Wekerle T. (2016). Kidney Transplantation With Corticosteroids Alone After Haploidentical HSCT From The Same Donor. Transplantation.

[21] Banff Foundation for Allograft Pathology (2022). Reference Guide to the Banff Classification. Banff Classification for Renal Allograft Pathology. https://banfffoundation.org/central-repository-for-banff-2019-resources-3/.

[22] Poggio E.D. (2019). Imaging as a Noninvasive Tool for Evaluating Interstitial Fibrosis in Kidney Allografts. Clin. J. Am. Soc. Nephrol..

[23] Belostotsky R., Frishberg Y. (2022). Catabolism of Hydroxyproline in Vertebrates: Physiology, Evolution, Genetic Diseases and New siRNA Approach for Treatment. Int. J. Mol. Sci..

[24] Schnackenberg L.K., Sun J., Pence L.M., Bhattacharyya S., da Costa G.G., Beger R.D. (2012). Metabolomics evaluation of hydroxyproline as a potential marker of melamine and cyanuric acid nephrotoxicity in male and female Fischer F344 rats. Food Chem. Toxicol..

[25] Montoro-Molina S., López-Carmona A., Quesada A., O’valle F., Martín-Morales N., Osuna A., Vargas F., Wangensteen R. (2018). Klotho and Aminopeptidases as Early Biomarkers of Renal Injury in Zucker Obese Rats. Front. Physiol..

[26] Can N., Alagöz A. (2020). The Relationship Among Bone Mineral Density, Bone Biomarkers and Vitamin D Levels in Patients with Parkinson’s Disease. Clin. Lab..

[27] Uguru C.C., Onwuka C.I., Obiechina A.E. (2021). Evaluation of urinary hydroxyproline and creatinine level in patients with benign mandibular odontogenic tumor. Clin. Exp. Dent. Res..

[28] Mavrogeorgis E., Mischak H., Latosinska A., Vlahou A., Schanstra J.P., Siwy J., Jankowski V., Beige J., Jankowski J. (2021). Collagen-Derived Peptides in CKD: A Link to Fibrosis. Toxins.

[29] Haddad R.G., Couranz S., Aviolp L.V. (1970). Nondialyzable Urinary Hydroxyproline as an Index of Bone Collagen Formation. J. Clin. Endocrinol. Metab..

[30] Mengel M., Reeve J., Bunnag S., Einecke G., Jhangri G.S., Sis B., Famulski K., Guembes-Hidalgo L., Halloran P.F. (2009). Scoring total inflammation is superior to the current banff inflammation score in predicting outcome and the degree of molecular disturbance in renal allografts. Am. J. Transplant..

[31] Marx D., Anglicheau D., Caillard S., Moulin B., Kochman A., Mischak H., Latosinska A., Bienaimé F., Prié D., Marquet P. (2023). Urinary collagen peptides: Source of markers for bone metabolic processes in kidney transplant recipients. Proteom. Clin. Appl..

[32] Zhou H., Yuen P., Pisitkun T., Gonzales P., Yasuda H., Dear J., Gross P., Knepper M., Star R. (2006). Collection, storage, preservation, and normalization of human urinary exosomes for biomarker discovery. Kidney Int..

[33] Jayachandran M., Lugo G., Heiling H., Miller V.M., Rule A.D., Lieske J.C. (2015). Extracellular vesicles in urine of women with but not without kidney stones manifest patterns similar to men: A case control study. Biol. Sex Differ..

[34] van Balkom B.W., Pisitkun T., Verhaar M.C., Knepper M.A. (2011). Exosomes and the kidney: Prospects for diagnosis and therapy of renal diseases. Kidney Int..

[35] Quesada A., Segarra A.B., Montoro-Molina S., Gracia M.d.C.d., Osuna A., O’valle F., Gómez-Guzmán M., Vargas F., Wangensteen R. (2017). Glutamyl aminopeptidase in microvesicular and exosomal fractions of urine is related with renal dysfunction in cisplatin-treated rats. PLoS ONE.

